# A new species of Turbanellidae (Gastrotricha, Macrodasyida) from Jamaica, with a key to species of *Paraturbanella*

**DOI:** 10.3897/zookeys.734.23023

**Published:** 2018-02-05

**Authors:** Matteo Dal Zotto, Francesca Leasi, M. Antonio Todaro

**Affiliations:** 1 Department of Life Sciences, University of Modena and Reggio Emilia, via Campi 213/d, I-41125 Modena, Italy; 2 Consortium for the Interuniversity Center of Marine Biology and Applied Ecology, viale N. Sauro 4, I-57128 Livorno, Italy; 3 Department of Invertebrate Zoology, Smithsonian National Museum of Natural History, 10th St. & Constitution Ave NW, Washington, D.C. 20560, United States

**Keywords:** Benthos, biodiversity, Caribbean Sea, meiofauna, taxonomy

## Abstract

The study falls within the framework of a wider research programme aimed at investigating the gastrotrich diversity of the Tropical North-Western Atlantic (TNWA). A new macrodasyidan gastrotrich is described from fine-medium sand collected at Duncans Bay, Jamaica. The description is based on observations carried out on living specimens using differential interference contrast microscopy. *Paraturbanella xaymacana* **sp. n.**, the third gastrotrich taxon reported from Jamaica, is a mid-sized species, up to 564 μm long, with a feeble peribuccal swelling. The most obvious autapomorphic traits pertain to the testes and the male pore, both of which are located approximately at mid body, rather than at- or near the pharyngo-intestinal junction as occur in the other species of the genus. Additional differences with congeners are discussed and a key to the *Paraturbanella* species is provided, in the hope it will be useful to both gastrotrich experts and marine ecologists who discover these microscopic metazoans during their research.

## Introduction

The biodiversity of microscopic organisms belonging to meiofauna is scarcely known compared to other metazoans. Knowledge is particularly scarce for the ‘minor phyla’, such as Kinorhyncha (Dal Zotto 2015, Sørensen et al. 2015, Dal Zotto and Todaro 2016) or Gastrotricha (Todaro et al. 2011, 2015). Gastrotricha includes microscopic, vermiform invertebrates found in both freshwater and marine ecosystems (see Kieneke and Schmidt-Rhaesa 2014). As of December 2017, the group comprises 840 species divided into the two orders Macrodasyida and Chaetonotida (Todaro 2017). Macrodasyida generally includes taxa living interstitially in marine sandy bottoms (but see e.g., Todaro et al. 2012), while Chaetonotida comprises species found from marine to freshwater environments. The alpha biodiversity and systematics of the Phylum are changing at a fast pace, as shown by the continuous finding and description of new taxa (for marine taxa, e.g., Hochberg et al. 2014, Todaro et al. 2014, 2015, Lee and Chang 2017) and the in-group phylogenetic reassessments (e.g., Kånneby et al. 2013, Todaro et al. 2014, Kånneby and Todaro 2015).

The present study is part of a larger research programme aimed at shedding light on the diversity and phylogeny of gastrotrich species of the Tropical North-Western Atlantic. From 2010 to 2013, several international groups of researchers surveyed the gastrotrich fauna of different islands in the South Floridian, Bahamian, Lesser Antilles and Central Caribbean ecoregions. Accounts of these studies can be found in, e.g., Hochberg and Atherton (2010, 2011), Hummon (2010a), Atherton and Hochberg (2012a, b), Hochberg et al. (2013, 2014), Atherton (2014), Kånneby et al. (2014), Von Und Zu Gilsa et al. (2014), Kieneke et al. (2015), Araujo and Hochberg (2017a, b), Schuster et al. (2017). Research teams headed by one of us (MAT) have visited three islands: St. John in the US-Virgin islands, Jamaica, and Curaçao. Part of the information and/or taxa found have appeared in several papers (e.g., Hummon et al. 2010, Kånneby et al. 2012, 2013, 2014, Todaro et al. 2012, Schuster et al. 2017). Specifically devoted to the Jamaican survey were two papers dealing with the description a new species of *Macrodasys* and the description of a new species, genus and family (Todaro and Leasi 2013, Todaro et al. 2014).

We describe here a new species of *Paraturbanella* from the northern shore of Jamaica. It shares the same position of the male gonads with a recently described species from South Africa. In addition, we propose a determination key to the species of the genus.

## Methods

Sampling campaign took place in February 2011 and included 10 locations along the North and West coasts of Jamaica. The species described herein was found in samples collected by hand from the shallow sublittoral (- 0.5 m); about 1 L of sandy sediment was placed into 500 mL plastic jars (Todaro 2002) and soon after brought to the field laboratory (Discovery Bay Marine Laboratory). The specimens were extracted daily with the narcotisation-decantation technique using a 7 % magnesium chloride solution, within one week from collection. The supernatant was poured, without filtering, into plastic Petri dishes (3.0 cm diameter) and scanned for gastrotrichs at max. 50 × magnification under a Wild 3 stereomicroscope (Todaro and Hummon 2008).

The gastrotrich specimens of interest were picked out with a micro-pipette, mounted on glass slides in a drop of 7 % MgCl_2_ solution, and studied in vivo with Nomarski differential interference contrast optics using a Zeiss Axio Scope A1. Photographs and measurements were taken with a DS-5M Nikon digital camera and Nikon NIS-F software. The description of the new species follows the convention of Hummon et al. (1993), whereas the position of some morphological characteristics along the body are given in percentage units (U) of total body length measured from anterior to posterior ends.

Abbreviations used in the text are as follows: PhIJ, pharyngo-intestinal junction; TbA, adhesive tubes of the anterior series; TbD, adhesive tubes of the dorsal series; TbDL, adhesive tubes of the dorsolateral series; TbL, adhesive tubes of the lateral series; TbP, adhesive tubes of the posterior series; TbV, adhesive tubes of the ventral series; TbVL, adhesive tubes of the ventrolateral series.

Granulometric analysis of the substrata was carried out according to Todaro et al. (2006). Mean grain size, sorting coefficient, kurtosis, and skewness were calculated by a computerised programme based on the equation of Seward-Thompson and Hails (1973).

Frequency of a species within collected samples follows Hummon et al. (1992) and is denoted as: 1) Sparse, when a species is found in less than 10 % of samples; 2) Occasional when found in 10–30 % of samples; 3) Common, in 30–60 % of samples; and 4) Usual, in more than 60 % of samples. Abundance of a species within a sample is classified as: 1) Rare, when contributing less than 1 % of a sample; 2) Scarce, 3–5 % of a sample; 3) Numerous, 10–20 % of a sample (often a sub-dominant); and 4) Prevalent, more than 30 % of a sample (usually dominant or co-dominant).

In the identification key we consider as ventrolateral the adhesive tubes that in some instances have been called, by other authors, lateral tubes. Furthermore, we consider *Paraturbanella dolichodema* Hummon, 2010 furnished with dorsal adhesive tubes and lacking ventral adhesive tubes, contra the original description that indicated that dorsal tubes are absent and the ventral tubes are present (Hummon 2010b). Our choice is based on information derived from the video sequences of the species made available from the original author (see especially vid5 at http://www.gastrotricha.unimore.it/moviegallery.htm). It should be noted that in *Paraturbanella*, ventral adhesive tubes have been reported only for *P. dolichodema*. This fact, and the position of the tubes described as originating in-between the ventral locomotory cilia raised our initial concern.

## Taxonomic account

### Phylum Gastrotricha Metschnikoff, 1865

#### Order Macrodasyida Remane, 1925 [Rao & Clausen, 1970]

##### Family Turbanellidae Remane, 1926

###### Genus *Paraturbanella* Remane, 1927

####### Paraturbanella xaymacana sp. n.

Taxon classificationAnimaliaMacrodasyidaTurbanellidae

http://zoobank.org/5E38C61A-5233-4E8E-8092-45E1B9AE000E

Figs 1 , 2 , 3

######## Type locality.

The sediment samples were collected on 24 February 2011 from Duncans Bay, Duncans, Jamaica (18°29'13.05'N, 77°32'03.23"W).

######## Type specimen.

Holotype: the 542 μm long adult specimen shown in Figures 2, 3, no longer extant (International Code of Zoological Nomenclature, Articles 73.1.1 and 73.1.4), collected on 24 February 2011 (MAT & FL legit).

######## Examined material.

Two adults (including the holotype) collected by MAT & FL from the type locality; specimens were observed alive and are no longer extant i.e., both physical specimens were inadvertently destroyed during the study. Considering the size and nature of these organisms, the provided drawings, and the original multiple photos of the studied animals, the establishment of a new species-group taxon should be considered valid under the recommendation 73G-J of Declaration 45 – Addition of Recommendations to Article 73 (ICZN 2017).

######## Ecology.

Sparse in frequency of occurrence (10 % of samples), scarce in abundance (3–5% of a sample); sub-littoral at a water depth of about 0.5 m in sediment made up of fine, moderately sorted carbonate sand (mean grain size, 0.18 mm; sorting 0.59; kurtosis, 2.52; skewness, 0.43). Values of salinity and temperature of the interstitial water at the time of sampling were 34 ‰ and 26 °C respectively.

######## Diagnosis.

Body strap-shaped, up to 564 μm in length. Head with a feeble peribuccal swelling, with a slight constriction at U3.7; pestle organs present. PhJIn at U31; body widest from mid-pharynx to mid-intestine, thinning gradually to the caudal base; caudum bilobed, incised from its tips to U95, with a clearly visible medial cone; distance between apices of outermost TbP on either side is 1.3 times the width of the caudal base. About 20–23 glands are distributed along both lateral body margins in a single column per side. TbA six per side, the innermost being the shortest, whereas the adjacent being the longest, occur on fleshy hands that insert at approximately U11; TbV, TbVL, TbL and TbD absent; TbP, six per side, occurring as 4, 1, 1, the outermost being the longest; caudal cone present; accessory adhesive tubes (called also dohrni/Seitenfüsschen) two per side, posterolaterally directed (longer tube = 21 μm, shorter tube = 14 μm), inserting ventrolaterally just behind the hands at U14. Locomotor ciliature runs from the TbA rearward in two longitudinal bands that trace the lateral body margins, joining after the anus. Mouth terminal, width narrow; buccal cavity medium-sized, mug-shaped; walls heavily cuticularized; pharyngeal pores near the base at U28; intestine straight, broadest in front; anus ventral at U91. Hermaphroditic, paired testes extend rearward from U51, with sperm ducts recurving to the fore at U63 and emptying to the exterior via a common pore at U49; paired ovaries, the largest ovum occurs in the mid-gut region at U51. Frontal organ dorsal to the intestine at U63.

######## Etymology.

The specific name alludes to the original name of Jamaica: *Xaymaca*, (adjective: xaymacana) an Arawak word meaning “land of wood and water”.

######## Description.

Mostly based on the adult holotype, 542 μm in total length. Body strap-shaped; head with a feeble peribuccal swelling and a slight constriction at U04 and then the body proper. Pestle organs, small, at U5; body widest at mid-intestine, thinning gradually to the caudal base; caudum bilobed, deeply incised from its tips to U95, with a visible medial cone; distance between apices of outermost TbP on either side is 1.3 times the width of the caudal base. Widths at outer oral opening/head constriction/mid-pharynx/PhJIn/mid-intestine/furcal base, and their locations along the body length are: 12/26/33/39/45/29 μm at U0/U04/U17/U31/U61/U95. Epidermal glands are in one column per side, scattered along the body margins, up to 20–23 and variable in size (4–7 μm in diameter).

*Adhesive tubes.* TbA, six per side (7–11 μm in length), all occurring on fleshy hands that insert at approximately U11; the innermost, mimicking a thumb, is the shortest, while the second from the inner side is the longest; TbV, TbVL,TbL, TbD absent; TbP, six per side, occurring as two groups of 4, 1, 1 elements each, along the inner (4 + 1 tube) and distal margin of each lobe (1 tube); the distal tube being the longest (14 μm in length) and the four proximal ones the shortest (6–7 μm in length); a caudal medial cone is present, but it is rather short, 4 μm in length. Accessory adhesive tubes (known also as dohrni tubes or Seitenfüsschen) two per side, posterolaterally directed (longer tube=21 μm, shorter=13.7 μm from their base), arise ventrolaterally just behind the fleshy hands at U14, usually being held close to the body.

*Ciliation.* Tufts of sparse cilia (11–21 μm in length) occur on lateral and dorsal sides of the head, behind the mouth. Additional sensory hairs, of similar length (13–19 μm), occur along the pharyngeal and intestinal region, organized in lateral, dorsolateral and dorsal columns, with about 20–23 hairs per column. Ventral locomotor cilia (16–20 μm in length) flow from the head constriction rearward in two longitudinal bands that trace the lateral body margins, and join behind the level of the anus.

*Digestive tract*. Mouth terminal, narrow (9 μm diameter); buccal cavity large, mug-shaped, 18 μm in length and approximately 11 μm in width, with walls heavily cuticularized; Pharynx 153 μm in length, with pharyngeal pores near the base at about U28; PhJIn at U31; intestine straight, broadest in front; anus ventral at U91.

*Reproductive tract*. Hermaphroditic; paired testes extend posteriorly from U51, with short sperm ducts recurving toward the front at U63, and emptying to the exterior via a common pore located at U49; ovaries paired, with the oocytes occurring from U64 to U68 and maturing from posterior to anterior; a large egg (approximately 70 by 24 μm) was present in the mid-gut region centred at U51. Caudal organ absent; frontal organ, vesicular, dorsal to the intestine centered at about U63; it is ovoid in shape (28 by 26 μm) and contains sparse spermatozoa and secretory material.

######## Variability and remarks.

**Figure 1.**
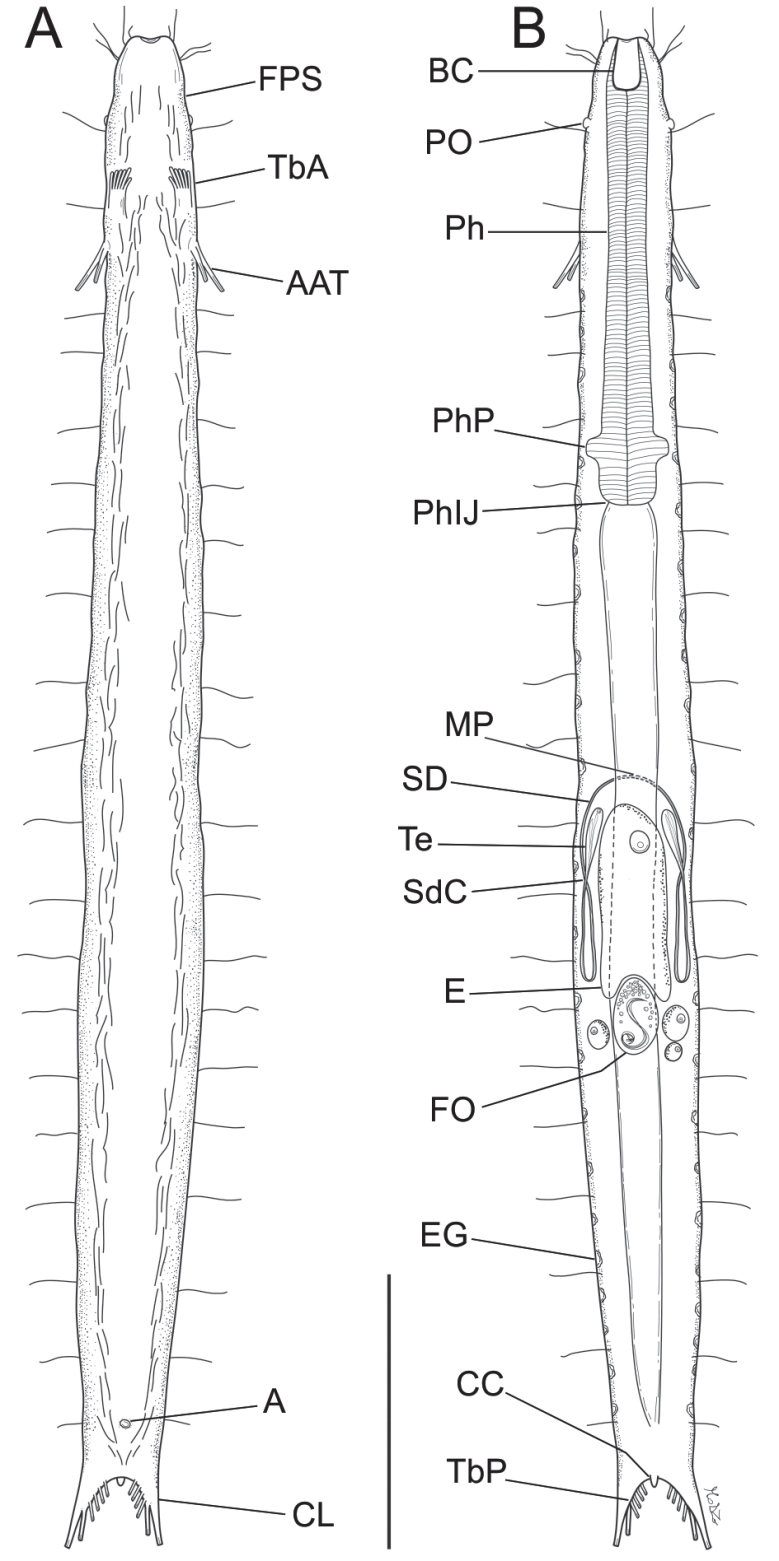
Line-art illustration of *Paraturbanella xaymacana* sp. n. **A** Habitus as seen from the ventral side **B** Habitus as seen from the dorsal side, showing the internal anatomy. Abbreviations: **A** anus **AAT** additional adhesive tubes (Seitenfüsschen) **BC** buccal cavity **CC** Caudal cone **CL** caudal lobe **E** egg **EG** epidermal gland **FO** frontal organ **FPS** Fleeble peribuccal swelling **MP** male pore **Ph** pharynx **PhIJ** pharyngo-intestinal junction **PhP** pharyngeal pore **PO** pestle organ **Sd** sperm duct **SdC** sperm duct crossing **TbA** anterior adhesive tubes **TbP** posterior adhesive tubes **Te** testis. Scale bar: 100 μm.

The other studied adult specimen was 564 μm in total body length, with 154 μm long pharynx. Number and arrangement of TbA, and of the TbP along the caudal lobes matched those of the holotype. The placement of the testes and the male pore is similar to that of the holotype. Unfortunately the animal got destroyed during the study so no further details could be acquired. The unfortunate event happened while we were trying to confirm the crossing of the ascended and descendent tracts of the sperm ducts observed in the holotype (see Figures 1B, 3A), a trait never recorded before in Gastrotricha. Future studies could indicate whether the crossing is an autapomorphic character of the species or just a feature of the holotype.

######## Taxonomic affinities.

Prior to the current study there were 22 described species of *Paraturbanella* (Hummon 2010b, 2011, Hummon and Todaro 2010, Todaro et al. 2017). *P. xaymacana* sp. n., in virtue of its testes, located at about mid body instead than at- or near the PhIJ, approaches *P. africana* Todaro, Dal Zotto, Bownes & Perissinotto, 2017, recently described from the KwaZulu-Natal coast of South Africa (Todaro et al. 2017). These two species can easily been differentiated based on the following traits which, in our opinion, should be considered in order of importance: i) position of the male pore: located near the PhIJ in *P. africana* vs at about mid body in *P. xaymacana* sp. n.; ii) buccal swelling, very clear in *P. africana* vs almost non-existent in the new species; iii) TbA, number and arrangement: 5 tubes per side and without the innermost short “thumb” in the African species vs 6 tubes per side and with the shortest tube being the innermost one in the Jamaican species; iv) TbP, number and arrangement: 5 tubes, organized as 3, 1, 1 in *P. africana* vs 6 tubes organized as 4, 1, 1 in *P. xaymacana* sp. n.

**Figure 2.**
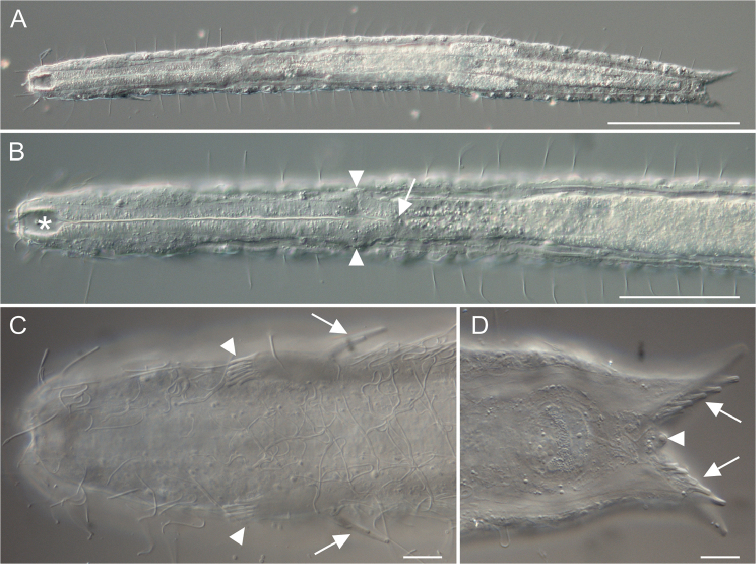
*Paraturbanella xaymacana* sp. n., holotype. Differential interference contrast photomicrographs. **A** Habitus, ventral view **B** Anterior region, ventral view, showing the buccal cavity (asterisk), the pharyngeal pores (arrowheads), and the pharyngo-intestinal junction (arrow) **C** Anterior region, ventral view, showing the lateral and ventral ciliation, the anterior adhesive tubes (arrowheads), and the additional adhesive tubes (Seitenfüsschen) (arrows) **D** Posterior region, ventral view, showing the medial cone (arrowhead) and the posterior adhesive tubes (arrows). Scale bars: 100 μm (**A**), 50 μm (**B**), 20 μm (**C–D**).

**Figure 3.**
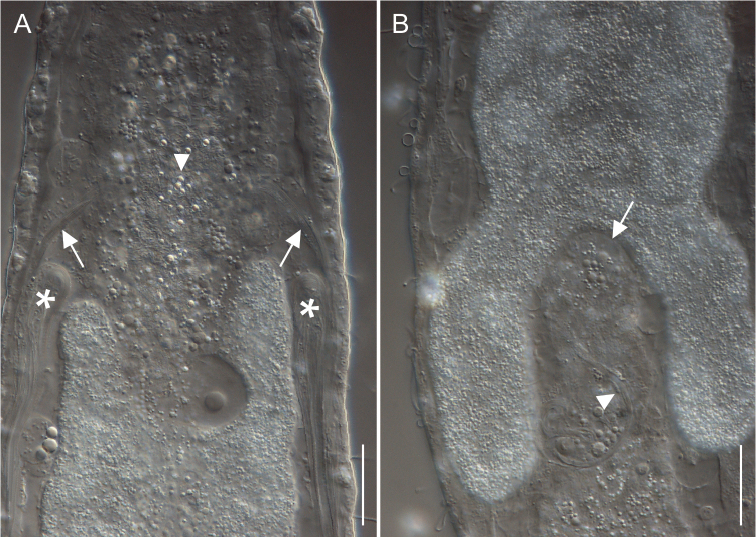
*Paraturbanella xaymacana* sp. n., holotype. Differential interference contrast photomicrographs. **A** Mid body, dorsal view, showing the testes (asterisks) beside a ripe egg, the sperm ducts (arrows), and the position of the male pore (arrowhead) **B** Mid body, dorsal view, showing the frontal organ (arrow) and a cluster of sperm (arrowhead). Scale bars: 20 μm (**A–B**).

### Taxonomic key

Several taxonomic keys to species of Gastrotricha have been developed in the last two decades (e.g. Todaro 2002, 2012, Clausen 2004, Kånneby et al. 2009, Todaro et al. 2009, Von Und Zu Gilsa et al. 2014, Garraffoni and Melchior 2015, Kieneke et al. 2015, , Kånneby 2016, Minowa and Garraffoni 2017). However, none of them have dealt with species of *Paraturbanella*. The tabular key of Clausen (1996) is of some utility but at least one species has been omitted (e.g., *P. brevicaudata* Rao, 1991) and several others have been described in the meanwhile. In marine habitats, the genus *Paraturbanella* is one of the most species rich and widespread; consequently, we hope the new key will prove useful not only to gastrotrich specialists but also to marine ecologists who find these peculiar metazoans in the course of research on interstitial meiobenthos. We warn the readers to refer to the original descriptions of the species, especially if the keyed-out taxa fall outside of their known geographic range of occurrence (see Table 1).

**Table 1.** Described species of *Paraturbanella* and their distribution.

Taxon	Distribution
*Paraturbanella africana*	KwaZulu-Natal, South Africa
*Paraturbanella aggregotubulata*	Florida, USA
*Paraturbanella armoricana*	Bretagne, France
*Paraturbanella boadeni*	Andaman, India
*Paraturbanella brevicaudata*	Lakshadweep, India
*Paraturbanella cuanensis*	Ireland and UK
*Paraturbanella dohrni*	Throughout the North Sea and the Mediterranean Sea; Gulf and Atlantic* coast of Florida, US; Red Sea*; Somalia (as P. cf dohrni)
*Paraturbanella dolichodema*	Pacific coast of the US
*Paraturbanella eireanna*	North Ireland
*Paraturbanella intermedia*	Washington State, US
*Paraturbanella levantia*	East Mediterranean Sea
*Paraturbanella manxensis*	Isle of Man, UK
*Paraturbanella mesoptera*	Andhra Pradesh, India
*Paraturbanella pacifica*	Galapagos Islands, Ecuador
*Paraturbanella pallida*	Throughout the Mediterranean Sea; Isles of Scilly, UK; Hawaii*.
*Paraturbanella palpibara*	Andhra Pradesh, India
*Paraturbanella pediballetor*	British Isles; Normandy, France
*Paraturbanella sanjuanensis*	Washington State, US
*Paraturbanella scanica*	Norway
*Paraturbanella solitaria*	Pacific coast of the US
*Paraturbanella stradbroki*	Queensland, Australia; Hawaii*
*Paraturbanella teissieri*	Throughout the North Sea and the Mediterranean Sea; Gulf and Atlantic coast of Florida, US

*, WD Hummon, personal communication.

### Taxonomic key to genus *Paraturbanella*

**Table:** 

1	TbVL present	**2**
–	TbVL absent	**10**
2	TbD present	**3**
–	TbD absent	**8**
3	TbD and TbVL clustered in the mid trunk region	***P. aggregotubulata* Evans, 1992**
–	TbD and TbVL uniformely distributed along the trunk region	**4**
4	TbP in a single row per side	**5**
–	TbP in a double row per side	***P. armoricana*** (**Swedmark, 1954a**)
5	Mouth protruding outwardly; testes just behind the PhIJ	**6**
–	Mouth not protruding outwardly; testes at some distance from the PhIJ	**7**
6	Head slightly narrowing forward; pestle organs faint; caudal cone elongate	***P. dolichodema* Hummon, 2010**
–	Head deeply narrowing forward; pestle organs prominent; caudal cone usually not presen	***P. levantia* Hummon, 2011**
7	Head slanted anteriorly; pestle organs and caudal cone absent	***P. stradbroki* Hochberg, 2002**
–	Head not slanted anteriorly; pestle organs and caudal cone present	***P. scanica* Clausen, 1996**
8	TbP in a single row per side	**9**
–	TbP in a double row per side	***P. manxensis* Hummon, 2008**
9	TbA, 5–6 per side; TbP, 7 per side	***P. eiranna* Maguire, 1976**
–	TbA, 11–12 per side; TbP, 10–13 per side	***P. intermedia* Wieser, 1957**
10	Head with a peribuccal swelling	**11**
–	Head without a peribuccal swelling	**17**
11	Head bearing anteriorly two pairs of club-shaped sensory palps	***P. palpibara* Rao & Ganapati, 1968**
–	Head lacking sensory palps	**12**
12	Testes at or near the PhIJ	.**13**
–	Testes at or passed mid body	.**16**
13	Head bearing ventral papillae	***P. teissieri* Swedmark, 1954b**
–	Head lacking ventral papillae	**14**
14	TbA, less than 8 per side	***P. solitaria* Todaro, 1995**
–	TbA, 8 or more per side	**15**
15	TbP, 8 per side occurring in pairs; caudal cone elongate	***P. sanjuanensis* Hummon, 2010**
–	TbP, 8–10 evenly spaced, caudal cone short	***P. mesoptera* Rao, 1970**
16	Peribuccal swelling noticeable; testes at mid body; TbA, 6 per side; TbP, 5 per side arranged as 3, 1, 1 elements	***P. africana* Todaro, Dal Zotto, Bownes & Perissinotto, 2017**
–	peribuccal swelling feeble ; testes passed mid body; TbA, 6 per side; TbP, 6 per side arranged as 4, 1, 1 elements	***P. xaymacana* sp**. **n.**
17	TbP in a single row per side	**18**
–	TbP in a double row per side	**22**
18	Total body length > 860 µm; caudal cone absent	***P. pediballetor* Hummon, 2008**
–	Total body length < 760 µm; caudal cone present	**19**
19	TbA inserted on the outer side of a cuticular rod	***P. boadeni* Rao & Ganapati, 1968**
–	TbA inserted in hand-like fashion on a fleshy base	**20**
20	Pestle organs absent	***P. cuanensis* Maguire, 1976**
–	Pestle organs present	**21**
21	Caudal lobes short and reduced; TbA, 6 per side; TbP, 5 per side	***P. brevicaudata* Rao, 1991**
–	Caudal lobes normally developed; TbA, 5–6 per side; TbP, 5–8 per side	. ***P. dohrni* Remane, 1927**
22	Total body length < 400 µm; pestle organs absent	***P. pacifica* Schmidt, 1974**
–	Total body length > 600 µm; pestle organs present	***P. pallida* Luporini, Magagnini & Tongiorgi, 1971**

## Supplementary Material

XML Treatment for Paraturbanella xaymacana
